# Development of a wheelchair mobility skills test for children and adolescents: combining evidence with clinical expertise

**DOI:** 10.1186/s12887-017-0809-9

**Published:** 2017-02-13

**Authors:** Marleen Elisabeth Sol, Olaf Verschuren, Laura de Groot, Janke Frederike de Groot, Fit-For-the-Future!-consortium

**Affiliations:** 1 Research Group Lifestyle and Health, HU University of Applied Sciences Utrecht, Heidelberglaan 7, Postbus 12011, Utrecht, 3501 AA The Netherlands; 2 Child Development and Exercise Center, Wilhelmina Children’s Hospital, University Medical Center Utrecht, Utrecht, The Netherlands; 3 Brain Center Rudolf Magnus and Center of Excellence for Rehabilitation Medicine, University Medical Center Utrecht, Utrecht, The Netherlands; 4 De Hoogstraat Rehabilitation, Utrecht, The Netherlands

**Keywords:** Children, Wheelchair mobility skills, Wheelchair mobility, Outcome measure

## Abstract

**Background:**

Wheelchair mobility skills (WMS) training is regarded by children using a manual wheelchair and their parents as an important factor to improve participation and daily physical activity. Currently, there is no outcome measure available for the evaluation of WMS in children. Several wheelchair mobility outcome measures have been developed for adults, but none of these have been validated in children. Therefore the objective of this study is to develop a WMS outcome measure for children using the current knowledge from literature in combination with the clinical expertise of health care professionals, children and their parents.

**Methods:**

Mixed methods approach. Phase 1: Item identification of WMS items through a systematic review using the ‘COnsensus-based Standards for the selection of health Measurement Instruments’ (COSMIN) recommendations. Phase 2: Item selection and validation of relevant WMS items for children, using a focus group and interviews with children using a manual wheelchair, their parents and health care professionals. Phase 3: Feasibility of the newly developed Utrecht Pediatric Wheelchair Mobility Skills Test (UP-WMST) through pilot testing.

**Results:**

Phase 1: Data analysis and synthesis of nine WMS related outcome measures showed there is no widely used outcome measure with levels of evidence across all measurement properties. However, four outcome measures showed some levels of evidence on reliability and validity for adults. Twenty-two WMS items with the best clinimetric properties were selected for further analysis in phase 2. Phase 2: Fifteen items were deemed as relevant for children, one item needed adaptation and six items were considered not relevant for assessing WMS in children. Phase 3: Two health care professionals administered the UP-WMST in eight children. The instructions of the UP-WMST were clear, but the scoring method of the height difference items needed adaptation. The outdoor items for rolling over soft surface and the side slope item were excluded in the final version of the UP-WMST due to logistic reasons.

**Conclusions:**

The newly developed 15 item UP-WMST is a validated outcome measure which is easy to administer in children using a manual wheelchair. More research regarding reliability, construct validity and responsiveness is warranted before the UP-WMST can be used in practice.

**Electronic supplementary material:**

The online version of this article (doi:10.1186/s12887-017-0809-9) contains supplementary material, which is available to authorized users.

## Background

Two of the most common motor disorders in childhood in the Netherlands are Cerebral Palsy with a prevalence of 2.5 per 1000 births [1], and neural tube defects with a prevalence of 6.52 per 10.000 births [2]. A large proportion of these children use a manual wheelchair for their daily mobility [3]. In adults, several studies have reported on the importance of wheelchair mobility skills (WMS) to overcome mobility problems and improve participation [4, 5]. Moreover, it has been shown that WMS training in adults can decrease their mobility problems by improving their WMS [6–9]. In children, evidence is limited, with only one pilot study by Sawatzki et al. looking at the effects of WMS training in six children using a manual wheelchair [10]. At the same time though, the importance of WMS training in children was recently confirmed in a qualitative study exploring factors associated with levels of physical activity [11]. One of the facilitating factors identified by children and their parents was WMS training. This can be illustrated by a quote from one of the parents: *“Wheelchair training, that is very important I think, .she can do much more now….a lot of places are not adjusted for wheelchairs ….and you can just go….your life becomes a lot more fun”* [11].

In the last decade a large variety of WMS related outcome measures has been developed for adults using a manual wheelchair [12]. In order to evaluate a WMS training for children, there is a need for such an outcome measure in this population as well. The pilot study by Sawatzki et al. was the only intervention study reporting on the use of a WMS outcome measure in children and used an adapted version of the WST 3.2 [10]. However, this WMS outcome measure was developed for adult manual wheelchair users and has not been validated for use in children. It is recommended to validate an outcomes measure again if it is applied in a new population [13]. This is important because certain items could be irrelevant, other items might need adaptation or new items need to be included for different populations. In this case wheelchair outcome measures have been developed for adults with spinal cord injury, stroke or amputation, whereas children more often use a manual wheelchair due to congenital defects such as cerebral palsy or neural tube defects.

To the best of our knowledge, no WMS outcome measure has been specifically developed for or validated in children using a manual wheelchair.

The best available WMS outcome measures for adults could potentially be used for validation in children. Unfortunately, there is currently no consensus among clinicians and researchers on the best outcome measure in adults to evaluate WMS [12, 14, 15]. One of the reasons for this lack in consensus could be the difference in definitions used for the selection of items, including wheelchair user function, manual wheelchair use, wheelchair driving or wheelchair mobility [12, 16]. In this paper we use the term WMS, as skills that address aspects of wheelchair mobility. In the International Classification of Functioning (ICF) (http://apps.who.int/classifications/icfbrowser/) wheelchair mobility is classified in chapter 4 (Mobility) as moving around using equipment (d465) and defined as “moving the whole body from place to place, on any surface or space, by using specific devices designed to facilitate moving or create other ways of moving around, such as a wheelchair”. This definition excludes other activities in a wheelchair such as transferring oneself or handling objects.

There is currently no outcome measure available for the evaluation of WMS training in children. Therefore, the objective of this study was to develop (based on available literature and expert opinion) a WMS outcome measure for children using a manual wheelchair.

## Methods

**Fig. 1:**
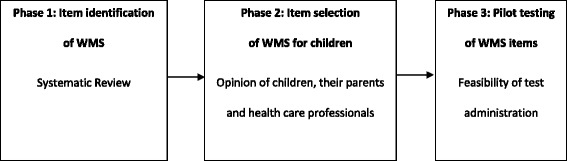
Methodological process of the development of a WMS outcome measure for children

In this study, the recommendations for the development of outcomes measures by the ‘COnsensus-based Standards for the selection of health Measurement Instruments’ (COSMIN) checklist [17] was followed. The COSMIN checklist was developed in a Delphi study by an international team of leading experts in epidemiology, psychometrics, and health care [17]. One of these recommendation involves combining evidence from literature with clinical expertise, i.e. opinion of the target population and health care professionals [13]. This process is illustrated in Fig. 1 and included the following phases: (1) Identification of potentially relevant WMS items with good measurement properties through a systematic review and best evidence synthesis regarding validity, reliability and responsiveness of existing WMS outcome measures (2) Selection of WMS items relevant for children using the opinion of children, their parents and health care professionals, (3) Pilot testing the feasibility of WMS items in children using a manual wheelchair.

### Phase 1 item identification of WMS

#### Data sources and searches

We updated the most recent systematic review on WMS from 2010 by Fliess-Douer et al. [12]. The same search string as Fliess-Douer et al. [12] was applied to the following databases: Pubmed, Cochrane and Web of Science up to July 2015. The full search strategy for Pubmed is described in Additional file 1.

#### Study selection

The selection of articles was independently performed by two reviewers (MS and JdG). While the search string was similar to Fliess-Douer et al. [12] the criteria used for selection were adapted to include WMS outcome measures for people with all types of disability, instead of only those for people with a spinal cord injury (SCI). This resulted in the following inclusion criteria : (1) aim of the study was to assess wheelchair skill performances in a wheelchair, (2) outcome measure is constructed for people using a manual wheelchair, (3) available statistical data regarding reproducibility or validity (4) full report written in English and publication date January 2010–July 2015. Studies were excluded when: (1) constructed for people using power wheelchairs, (2) developed for assessing in virtual environment, (3) focused on ‘body function and structures’ (measuring specific physiological and/or biomechanical variables which do not comply with the terms of ‘activity’ or ‘participation’ domains as defined in the ICF (http://apps.who.int/classifications/icfbrowser/).

#### Assessment of methodological quality

Studies reporting a total and item score were divided into sub studies to be able to differentiate between statistical methods being used. Two reviewers (MS and JdG) independently evaluated the methodological quality of the included studies using the COSMIN checklist [18]. The COSMIN checklist contains twelve boxes, which assess the methodological quality of the studies regarding reliability, measurement error, content validity, hypothesis testing, cross-cultural validity, structural validity, criterion validity, and responsiveness. The items in each box are rated with a 4-point scoring system; excellent, good, fair, and poor. A quality score per measurement property was obtained by taking the lowest rating of any item in a box (“worst score counts”). One item in each box concerns the sample size requirements, with a minimal requirement of *n* > 30 for an adequate sample size. As the COSMIN checklist was originally developed for health related questionnaires, sample size requirements might differ for performance based measures and can alternatively be based on power calculations as earlier discussed by Bartels et al. [19]. Therefore, the sample size requirement for assessment of methodological quality of reliability was adjusted to N ≥ 20, based on a sample size determination for a WMS outcome measure with power calculation from Kirby et al. [20].

#### Data extraction and best evidence synthesis

Two reviewers (MS, OV) independently performed the data extraction and assessed the results of the studies based on the quality criteria described by Terwee et al. [21]. The possible ratings per measurement property were “positive,” “indeterminate” and “negative”. Studies looking at different measurement properties of the same outcome measure were pooled for best evidence synthesis. This synthesis combines the methodological quality of the studies with the consistency of their results [22]. The level of evidence for each outcome measure was subsequently rated as “strong”, “moderate”, “limited”, “conflicting”, or “unknown” per measurement property. This method is similar to the method used for the systematic review of clinical trials as suggested by the Cochrane Collaboration Back Review Group [22].

#### Selection of WMS outcome measure

The WMS outcome measures with some level of evidence across reliability and validity were grouped together for item selection in phase 2.

### Phase 2: item selection of WMS for children

The resulting list of WMS items identified in phase 1 was assessed on their relevance for children using a manual wheelchair. Relevance checking was performed through a focus group or individual interviews with children using a manual wheelchair, their parents and health care professionals. The children and their parents were recruited from a voluntary WMS training program, which was set to start a few weeks later. Physiotherapy students were trained by an experienced qualitative researcher to conduct interviews with parents and children following a topic list. Individual interviews were conducted with the children and their parents separately or, in case this was preferred by the child, together. The parents and children were asked open ended questions about their current limitations regarding wheelchair mobility, their expectations of the WMS training and training goals. Open ended interview questions were preferred over relevance checking per item as this method assured an open mind regarding WMS which are relevant for children, without being influenced by WMS for adults. All interviews were recorded by video and transcribed verbatim. After transcription, a qualitative Framework Method Analyses [23] was performed for all interviews by two independent researchers to determine relevant items. The coding framework was based on the compiled list of items from the results of phase 1. Concurrently with the individual interviews, a focus group interview was conducted with health care professionals with clinical expertise in pediatric rehabilitation. All health care professionals were currently working at a special needs school and employed by De Hoogstraat rehabilitation centre, the Netherlands. Every potential WMS item from phase 1 was assessed in the focus group with health care professionals on the appropriateness for children and rated as ‘relevant’ , ‘relevant with adaptations’ or ‘not relevant’. Professionals were asked to keep in mind a total test duration of an hour, to make sure all items were critically assessed on relevance. One researcher (LdG) documented the answers given by the professionals. The results of the qualitative framework analyses of the target population was combined with the opinion of the health care professional to develop a new assessment tool with the work name: Utrecht Pediatric Wheelchair Mobility Skills Test (UP-WMST).

### Phase 3: Pilot testing of WMS items

One occupational therapist and one physiotherapist were asked to provide written comments and answer question regarding: 1) the feasibility to assess WMS within one hour; 2) the ease of handling material; and 3) clarity of instructions when administering the UP-WMST to children using a manual wheelchair. This was followed by individual interviews with the therapists. Both health care professionals received a manual of the UP-WMST with instructions about test set-up and instructions per item. Children who use a manual wheelchair were recruited from a special needs school in Utrecht, the Netherlands.

## Results

### Phase 1: item identification of WMS

#### Search results

**Fig. 2:**
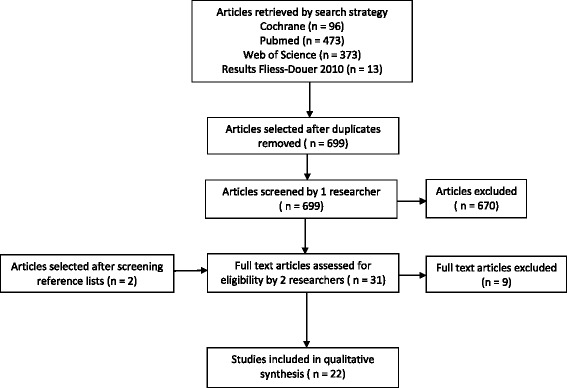
Flow chart of the search strategy till July 2015 and selection of articles

The search strategy combined with the previous results from Fliess-Douer et al. [12] resulted in a total of 699 unique articles, of which 31 were selected for full text assessment (Fig. 2). Nine studies were excluded after full text assessment. After exclusion, 22 studies were considered eligible for this review. The main reasons for exclusions were; the absence of psychometric properties of the outcome measure being used [10, 24–27]; outcome measures focused on the level of ‘body function and structures’ [25, 28] and one outcome measure was a questionnaire [29].

#### Study characteristics

The 22 studies reported on 15 different outcome measures. The general characteristics of these outcome measures are presented in Table 1. The Wheelchair Circuit [30–33] (WC) and the Wheelchair Skills test ([20, 34, 35], http://www.wheelchairskillsprogram.ca/eng/publications.php) (WST) have been further developed into additional versions. Most outcome measures were constructed to assess either wheelchair mobility (TOWM, Wheelie test, Wheelchair Circuit, HMAT and OCAWUP) [30–32, 36–41] or wheelchair user function (WST, AML, TMT, WUFA, WC-PFP, VFM) [20, 34, 35, 42–46]. Three outcome measures focused on a specific aspect of wheelchair mobility; wheelchair propulsion (WPT, slalom test) [47, 48] or wheelchair driving (WC-WAIMS) [33]. Two outcome measures were constructed to reflect a broad overview of physical function [49] and mobility [50]. All fifteen outcome measures contained items specifically related to wheelchair mobility, ranging from 1/11 WMS items [45] to 10/10 WMS [40, 41] items per total number of items.

**Table 1:** Characteristics of WMS outcome measures

Instrument		Study	Construct	Target population		# WMS items/# total items	Duration	Outcome
	Version			Disease characteristics	Age			Parameters (Scale)
WPT		Askari (2013) [47]	Manual wheelchair propulsion	Manual wheelchair user	≥17 years	1/1	≤ 50 s	Propulsion method
TOWM & Wheelie test		Fliess-Douer (2012, 2013a, 2013b) [36–38]	Manual wheelchair mobility	SCI	18–65 years	36/38	40 min	Ability (0–1), Quality (0–5), Time (s), Anxiety (0–10)
WC		Kilkens (2002, 2004) [30, 31]	Manual wheelchair mobility	SCI	18–65 years	5/8	na	Ability (0–1), Performance time (s), Physical strain (max HR)
	AM WC	Cowan (2011) [32]	Manual wheelchair mobility	SCI	> 18 years	12/14	na	Ability (0–1), Performance time (s)
	WAIMS	Vereecken (2012) [33]	Manual wheelchair driving	MS	na	7/8	20–30 min	Ability (0–2), Performance time (s), Covered distance (m)
Slalom test		Gagnon (2011) [48]	Manual wheelchair propulsion	SCI	≥ 18 years	1/1	17 min	Time (s)
WST	1.0	Kirby (2002) [34]	Manual wheelchair user function in daily life	Manual wheelchair user	≥ 18 years	14/33	29 min	Safety completion (0–2),
	2.4	Kirby (2004) [20]	Manual wheelchair user function	Manual wheelchair user	≥ 17 years	30/50	27 min	Ability (pass/fail/NA), GAS
	4.1	Lindquist (2010) [35]	Manual wheelchair user function	Manual wheelchair user	> 16 years	24/30	31 min	Ability (pass/fail). Safety (safe/unsafe)
HMAT		Harvey (1998) [39]	Manual wheelchair mobility	SCI	na	3/6	< 15 min	Level of assistance (1–6)
FIM + 5AML		Middleton (2006) [42]	Manual wheelchair user functional independence	SCI	18–65 years	3/5	na	Level of assistance (1–7)
OCAWUP		Routhier (2004, 2005) [40, 41]	Wheelchair mobility	Manual and power wheelchair user	18–65 years	10/10	60–90 min	Time (s), Degree of ease (0–3)
TMT		Chafetz (2004) [43]	Manual wheelchair user function	SCI	8–14 years	2/6	60–90 min	Time (s)
WUFA		Stanley (2003) [44]	Manual wheelchair user function	Manual wheelchair user	na	6/13	60–90 min	Level of independence (1–7)
WC-PFP		Cress (2002) [45]	Manual wheelchair user function	Manual wheelchair user	18–67 years	1/11	40 min	Speed (1/time)
VFM		Taricco (2000) [46]	Manual wheelchair user function	SCI	All age groups	9/65	30–50 min	Level of independence (1–5)
TOMP		Gans (1988) [49]	Physical function and motor performance	All disabilities	> 6 years	2/32	< 60 min	Assistance (0–4), Approach (0–1), Pattern (0–1), Proficiency (0–2)
TM		Jebsen (1970) [50]	Mobility	Locomotor disorders	> 20 years	2/31	60 min	Time (s)

*WMS* wheelchair mobility skills, *WPT* wheelchair propulsions test, *TMT* Timed Motor Test for wheelchair users, *AM-WC* adapted manual wheelchair circuit, *WC-PFP* wheelchair physical functional performance test, *TOWM* test of wheeled mobility, *TOMP* tufts assessment of motor performance, *HMAT* Harvey mobility assessment tool, *TM* measurement of time, *WC* wheelchair circuit, *WST* wheelchair skills test, *FIM* functional independence measure, *5-AML* five additional mobility and locomotor items, *OCAWUP* obstacle course assessment of wheelchair user performance, *WUFA* wheelchair user functional assessment, *VFM* Valutazione Funzionale Mielolesi, *WAIMS* wheelchair assessment instrument for people with multiple sclerosis, *NA* not applicable, *SCI* spinal cord injury, *MS* multiple sclerosis, *HR* heart rate, *GAS* Goal attainment scale

Table 2 shows the general characteristics of the studies; number of participants, disease characteristics, mean age and sex. Due to sample sizes requirements of N ≥ 20, eight studies [35, 40, 41, 43–45, 48, 50] were excluded for further data synthesis and analysis. This exclusion includes the TMT [43] which was the only outcome measure specifically developed for children.

**Table 2:** General information per study

Study	Instrument	Participants (N)	Disease characteristics	Mean age (mean years ± SD)	Sex (M/F)
Askari et al. 2013 [47]	WPT	*N* = 58^a^	Amputation, Spinal Cord Injury, Stroke, Traumatic Brain Injury, others	58 ± 17.9	35/13
Chafetz et al. 2004 [43]	TMT	*N* = 11	Spinal Cord Injury	10.5 ± 2.1	6/5
Cowan et al. 2011 [32]	AM WC	*N* = 50	Spinal Cord Injury	41.9 ± 13.4	42/8
Cress et al. 2002 [45]	WC-PFP	*N* = 18^a^	Multiple Sclerosis, Stroke, Spinal Cord Injury, Polio, Arthritis, Brain injury	49.4 ± 10.6	13/5
Fliess-Douer 2012 [37]	TOWM & Wheelie	*N* = 126	Spinal Cord Injury	na	91/35
Fliess-Douer 2013(a) [36]	TOWM & Wheelie	*N* = 30^a^	Spinal Cord Injury	38.8 ± 8.0	na
Fliess-Douer 2013(b) [38]	TOWM & Wheelie	*N* = 30^a^	Spinal Cord Injury	38.8 ± 8.0	na
Gagnon et al. 2011 [48]	Slalom test	*N* = 15	Spinal Cord Injury	40.7 ± 12.6	na
Gans 1988 [49]	TOMP	*N* = 40	Neurological disorder, musculoskeletal disorder	25.6 ± 19.5	14/26
Harvey et al. 1998 [39]	HMAT	*N* = 20	Spinal Cord Injury	45.6 ± 16.8	na
Jebsen et al. 1970 [50]	TM	*N* = 18	Stroke, Amputation, Peripheral neuropathy, Polio, Spinal Cord Injury, others	49.7 ± na	na
Kilkens et al. 2002 [30]	WC	*N* = 27	Spinal Cord Injury	34.7 ± 12.5	18/9
Kilkens et al. 2004 [31]	WC	*N* = 74	Spinal Cord Injury	40.5 ± 14.5	51/23
Kirby et al. 2002 [34]	WST 1.0	*N* = 24	Amputation, Stoke, Musculoskeletal disorder, Spinal Cord Injury, Neuromuscular disorder	59 ± 19	16/8
Kirby et al. 2004 [20]	WST 2.4	*N* = 298^a^	Amputation, Musculoskeletal, Spinal Cord Injury, Stroke, Able bodied	43.8 ± 22.5	158/140
Lindquist et al. 2010 [35]	WST 4.1	*N* = 11	Spinal Cord Injury, Stroke, other	42.1 ± 16.2	9/2
Middleton et al. 2006 [42]	FIM +5-AML	*N* = 39	Spinal Cord Injury	28 ± 6.5	32/7
Routhier et al. 2004 [40]	OCAWUP	*N* = 17	Spinal Cord Injury, Neuromuscular disorder, Stroke, Amputation	50.9 ± 12.1	10/7
Routhier et al. 2005 [41]	OCAWUP	na	na	na	na
Stanley 2003 [44]	WUFA	*N* = 5	Spinal Cord injury, Cerebral Palsy, Transverse myelitis	34.2 ± 10.3	4/1
Taricco 2000 [46]	VFM	*N* = 100	Spinal Cord Injury	37 ± na	77/23
Vereecken 2012 [33]	WAIMS	*N* = 50	Multiple Sclerosis	50 ± 12	30/20

*M* male, *F* female, ^a^Subset of participants differs per measurement property used for reliability sample, *na* no information available, *WPT* wheelchair propulsions test, *TMT* Timed Motor Test for wheelchair users, *AM-WC* adapted manual wheelchair circuit, *WC-PFP* wheelchair physical functional performance test, *TOWM* test of wheeled mobility, *TOMP* tufts assessment of motor performance, *HMAT* Harvey mobility assessment tool, *TM* measurement of time, *WC* wheelchair circuit, *WST* wheelchair skills test, *FIM* functional independence measure, *5-AML* five additional mobility and locomotor items, *OCAWUP* obstacle course assessment of wheelchair user performance, *WUFA* wheelchair user functional assessment, *VFM* Valutazione Funzionale Mielolesi, *WAIMS* wheelchair assessment instrument for people with multiple sclerosis

#### Measurement properties

The methodological quality and level of evidence of the studies are presented in Tables 3 and 4 for each measurement property, arranged per outcome measure. No studies assessed all measurement properties. Reliability and hypothesis testing were the most frequently reported properties. Different methods were used to assess inter-rater reliability; some studies used two raters to separately assess the same video recording, whereas other studies used two raters to separately administer the test. Only three studies [44, 45, 47] demonstrated levels of evidence on content validity. Criterion validity was not assessed as there is no gold standard available. Some studies reported on the Smallest Detectable Change or Limits of Agreement, but no studies calculated the Minimal Important Change needed to determine the level of evidence for the measurement error of an instrument. Therefore no levels of evidence were found for any of the outcome measures on criterion validity and interpretability.

**Table 3:** Methodological quality of measurement properties on reliability and best evidence synthesis

Instrument		Author		Reliability				
	Version			Internal consistency	Test-retest	Intra rater	Inter rater	Measurement error
WST								
	1.0	Kirby 2002 [34]						
			Substudy 1: total score	.	poor/indeterminate	fair/positive	fair/positive^a^	.
			Substudy 2: Item score	.	poor/indeterminate	poor/indeterminate	poor/indeterminate^a^	.
	2.4	Kirby 2004 [20]						
			Substudy 1: total score	.	good/positive	good/positive	good/positive^a^	.
			Substudy 2: Item score	.	poor/indeterminate	poor/indeterminate	poor/indeterminate^a^	.
	Level of evidence			na	moderate positive	moderate positive	moderate positive	na
WPT								
		Askari 2013 [47]		.	fair/positive	.	good/positive^a^	.
	Level of evidence			na	limited positive	na	moderate positive	na
WC								
		Kilkens 2002 [30]		.	good/positive	.	good/positive	.
		Kilkens 2004 [31]		.	.	.	.	.
	AM-WC	Cowan 2011 [32]		.	good/positive	.	.	good/indeterminate
	WAIMS	Vereecken 2012 [33]		.	fair/positive	.	fair/positive	.
	Level of evidence			na	strong positive	na	moderate positive	unknown
TOWM Wheelie					.			
		Fliess-Douer 2012 [37]		.	.	.	.	.
		Fliess-Douer 2013(a) [36]						
			Substudy 1: quality item score	.	poor/indeterminate	poor/indeterminate	poor/indeterminate^a^	good/indeterminate
			Substudy 2: other scores	.	good/positive	.	.	good/indeterminate
		Fliess-Douer 2013(b) [38]		.	.	.	.	.
	Level of evidence			na	moderate positive	unknown	unknown	unknown
TOMP								
		Gans 1988 [49]		.	.	.	fair/positive^a^	.
	Level of evidence			na	na	na	limited positive	na
HMAT								
		Harvey 1998 [39]		.	.	.	good/positive	.
	Level of evidence			na	na	na	moderate positive	na
FIM +5-AML								
		Middleton 2006 [42]		fair/positive	.	.	.	.
	Level of evidence			limited positive	na	na	na	na
VFM								
		Taricco 2000 [46]		good/positive	.	.	.	.
	Level of evidence			moderate positive	na	na	na	na

^a^Based on video rating

**Table 4:** Methodological quality of measurement properties on validity, responsiveness and best evidence synthesis

Instrument		Author		Validity			Responsiveness
	Version			Content validity	Structural validity	Hypothesis testing	
WST							
	1.0	Kirby 2002 [34]					
			Substudy 1: total score	fair/indeterminate	.	fair/positive	.
			Substudy 2: Item score	.	.	.	.
	2.4	Kirby 2004 [20]					
			Substudy 1: total score	.	.	fair/positive	.
			Substudy 2: Item score	.	.	.	.
	Level of evidence			unknown	na	moderate positive	na
WPT							
		Askari 2013 [47]		good/positive	good/indeterminate	good/positive	.
	Level of evidence			moderate positive	unknown	moderate positive	na
WC							
		Kilkens 2002 [30]		.	.	.	.
		Kilkens 2004 [31]		.	.	fair/positive	fair/positive
	AM-WC	Cowan 2011 [32]		.	.	good/negative	.
	WAIMS	Vereecken 2012 [33]		.	.	fair/positive	.
	Level of evidence			na	na	conflicting	limited positive
TOWM Wheelie							
		Fliess-Douer 2012 [37]		poor/indeterminate	.	.	.
		Fliess-Douer 2013(a) [36]					
			Substudy 1: quality item score	.	.	.	.
			Substudy 2: other scores	.	.	.	.
		Fliess-Douer 2013(b) [38]		poor/indeterminate	.	poor/indeterminate	.
	Level of evidence			unknown	na	unknown	na
TOMP							
		Gans 1988 [49]		.	.	.	.
	Level of evidence			na	na	na	na
HMAT							
		Harvey 1998 [39]		.	.	.	.
	Level of evidence			na	na	na	na
FIM +5-AML							
		Middleton 2006 [42]		.	fair/positive	fair/positive	fair/positive
	Level of evidence			na	limited positive	limited positive	limited positive
VFM							
		Taricco 2000 [46]		.	fair/indeterminate	poor/indeterminate	fair/indeterminate
	Level of evidence			na	unknown	unknown	unknown

*WST* wheelchair skills test, *WPT* wheelchair propulsions test, *WC* wheelchair circuit, *AM-WC* adapted manual wheelchair circuit, *WAIMS* wheelchair assessment instrument for people with multiple sclerosis, *TOWM* test of wheeled mobility, *TOMP* tufts assessment of motor performance, *HMAT* Harvey mobility assessment tool, *FIM* functional independence measure, *5-AML* five additional mobility and locomotor items, *VFM* Valutazione Funzionale Mielolesi, *na* no information available

##### Wheelchair Skills Test (WST)

The WST 1.0 was originally developed by Kirby et al. [34] consisting of 33 items measuring wheelchair user functional skills in daily life for adults using a manual wheelchair. Fourteen of these items assess WMS, the other items assess other activities in a wheelchair, such as transfers or handling objects. The level of evidence for content validity of this outcome measure is unknown. A number of items and the outcome parameter were adapted in the WST 2.4 by Kirby et al. [20]. The WST 2.4 demonstrated good methodological quality for the reliability of the total score. The scoring of individual items reached a poor methodological quality, due to statistical flaws. Overall the WST shows moderate levels of positive evidence on reliability of the total score, moderate positive levels of evidence for hypothesis testing and unknown or no information on the other measurement properties.

##### Wheelchair Propulsion Test (WPT)

Askari et al. [47] reported on the WPT, which is a quick test consisting of one WMS item measuring several parameters of wheelchair propulsion. This studies demonstrates limited to moderate levels of positive evidence on reliability. Moderate levels of positive evidence on content validity and hypothesis testing. Even though the structural validity showed good methodological quality, the level of evidence is unknown as the explained variance was not mentioned in the results.

##### Wheelchair Circuit (WC)

The WC was developed to measure wheelchair mobility of adult manual wheelchair users with a SCI [30, 31]. Most of the items assess WMS, with an additional assessment of wheelchair transfer and wheelchair endurance. Several items were later adapted in the Adapted Manual Wheelchair Circuit (AM-WC) [32] to facilitate widespread utilization. Vereecken et al. [33] focused on the driving skills, and adapted the WC into the Wheelchair Assessment Instrument for people with Multiple Sclerosis (WAIMS). No studies reported on content validity regarding the SCI or MS population. The methodological quality of reliability was rated fair to good with a moderate to strong level of positive evidence. There is conflicting evidence regarding hypothesis testing, and only limited positive evidence on responsiveness.

##### Test of Wheeled Mobility (TOWM) and Wheelie Test

Fliess-Douer et al. [36–38] demonstrated poor content validity. All 38 items, except the wheelchair transfer, assess WMS. Although a large sample size was used to create a list of essential WMS, there was no assessment if all items together comprehensively reflect the construct to be measured. The statistical method regarding the reliability of item quality scores was inadequate, however the method used for all other scores was appropriate. Therefore the level of positive evidence is moderate for test-retest reliability of all scores, except for the item quality scores. There is unknown or no level of evidence for all other measurement properties.

##### Tufts Assessment of Motor Performance (TOMP)

This assessment tool for functional motor skills in all disabilities was developed by Gans et al. [49]. The tool consists of 32 items in total with two items assessing WMS. This study demonstrated a limited level of positive evidence for inter rater reliability. No other measurement properties were assessed.

##### Harvey Mobility Assessment Tool (HMAT)

Harvey et al. [39] developed an outcome measure that could quantify the mobility of wheelchair dependent paraplegics. Three out of the six items assessed WMS. Information on measurement properties were only reported on inter rater reliability. The methodological quality of reliability was rated as good with a moderate level of positive evidence.

##### Functional Independence Measure (FIM) + 5 Additional Mobility and Locomotor items (5-AML)

Middleton et al. [42] developed an additional three WMS items and two other wheelchair items to improve the sensitivity of the FIM for people with a SCI. Although they do not show any evidence on content validity or test-retest reliability they are the only study who demonstrated limited levels of positive evidence for internal consistency, structural validity, hypothesis testing and responsiveness.

##### Valutazione Funzionale Mielolesi (VFM)

The VFM was developed in Italy by Tarrico et al. [46] and consists of 65 items of which nine items assess WMS. This study used one of the largest sample sizes of all studies in this review and demonstrated good methodological quality for internal consistency. No other aspects of reliability were mentioned. This study did not report on content validity. The other properties of validity were of unknown level of evidence due to poor methodological for hypothesis testing and fair methodological quality for structural validity without explaining the variance.

### Conclusion phase 1: item identification of WMS

There is no widely used WMS outcome measure with levels of evidence across all measurement properties e.g. validity, reliability and responsiveness. However, the WST [20, 34], WPT [47], WC [30–33] and 5AML [42] already showed some level of evidence on aspects of reliability and validity. The individual WMS items of these four outcome measures seem to be the best WMS items available from literature for validation in children. The WST, WPT, WC and 5AML were combined into an overall list of 22 unique WMS items, excluding items not related to mobility as defined by the ICF d465 (http://apps.who.int/classifications/icfbrowser/). The first column in Table 5 shows the compiled list of WMS items and the original outcome measures they were selected from.

**Table 5:** Combining evidence from literature of phase 1 with the clinical expertise of phase 2 and 3

Phase 1: wheelchair mobility skill items		Phase 2: selection of relevant items							Phase 3: Pilot testing
		Health care professional	Children and their parents					Item selected for phase 3	Opinion of health care professional
		Relevant items	Relevant items	Activities					
				Cross the road	Maneuver in crowded place	Maneuver in small rooms	Uneven surfaces		
1. Propulsion forward^a, b, c, d^	10–50 m	Yes	Yes	x	x	x	x	Yes	Include
2. Propulsion backwards^a^	5 m	Yes			x	x		Yes	Include
3. Rolls on soft surface^a, c^	2 m (gravel, mat, grass)	Yes	Yes				x	Yes	Include mat, exclude gravel and grass
4. Turns 90° while moving forward^a^	Left	Yes	Yes		x	x		Yes	Include
	Right								
5. Turns 90° while moving backward^a^	Left	Yes			x	x		Yes	Include
	Right								
6. Turns 180° in place^a^	Left	Yes			x	x		Yes	Include
	Right								
7. Parallel parking/maneuvers sideways^a^	Left	No						No	-
	Right								
8. Avoids moving obstacle^a^	Left	Adapted			x			Adapt into sudden stop	Include
	Right								
9. Opening/Closing a door^a, c^	Open toward	Yes	Yes					Yes	Include
	Open away								
10. Figure-of-8-shape^c^		Yes			x	x		Yes	Include
11. Holding a Wheelie^a, c^	0–30 s	No	Yes					Yes	Include
12. Propelling in a Wheelie forward^c^	3 m	No						No	-
13. Turns 180° in place in wheelie position^a^	Left	No						No	-
	Right								-
14. Slope ascent^a, c, d^	3–20%	Yes	Yes					Yes	Include, adapt scoring
15. Slope descent^a, d^	3–20%	Yes	Yes					Yes	Include, adapt scoring
16. Side Slope^a, c^		Yes	Yes					Yes	Exclude, material too big
17. Platform ascending^a, c, d^	2,5–15 cm	Yes	Yes^e^	x				Yes	Include, adapt scoring
18. Platform descending^a^	2.5–15 cm	Yes	Yes^e^	x				Yes	Include, adapt scoring
19. Doorstep^a, c^	1,2–4 cm	Yes	Yes^e^					Yes	Include, adapt scoring
20. Gets over pothole^a^	15 cm	No						No	-
21. Ascends stairs^a^		No	Yes					No	-
22. Descends stairs^a^		No						No	-
New item		Gutter, escalator					sidewalk	No	-

^a^Wheelchair Skills Test, ^b^Wheelchair Propulsion Test, ^c^Wheelchair Circuit, ^d^5 Additional Mobility and Locomotor items, ^e^with and without anti-tippers

### Results phase 2: item selection of WMS for children

Individual interviews took 30–60 min and were conducted with three girls, eight boys and their parents. The children’s age ranged from 6 to 13 years old. The group consisted of two children with cerebral palsy, seven children with spina bifida, one child with congenital sodium diarrhea and one child with congenital myeasthenic syndrome. Parents and children gave descriptions of different community activities in daily life in which the WMS of the child were inadequate or where they would like to improve on. Framework data analysis resulted in WMS which were literally part of the compiled list of potentially relevant items as can be seen in the fourth column of Table 5. For example, children would like to improve in their ability to go over a steep ramp or to go up and down a high or low curb. In addition, there were new codes developed for the coding framework to categorize recurring themes which could not be attributed to a single WMS item. The subsequent four columns in Table 5 show these categories: ‘crossing the road’ , ‘maneuver in crowded places’ or ‘small rooms’ and ‘propel over uneven surfaces’ and their match to existing WMS items or if not available a new WMS item.

The focus group conducted with health care professionals consisted of five occupational therapists and five physiotherapists, with an average age of 34.4 (SD = 7.8) years and 8.0 (SD = 4.8) years of experience in working with children in a wheelchair. All 22 potentially relevant items were assessed in the focus group. Most items were considered relevant for children, however six items were deemed not appropriate for a WMS outcome measure for children: ‘ascending or descending stairs’ , ‘propelling in a wheelie’ , ‘turn 180° in wheelie position left and right’ and ‘get over a pothole’. Total time of administration was considered important due to the extra instruction time and shorter attention span of children when administering an outcome measure. When considering these time restraints health care professionals suggested that while ‘holding a wheelie’ is a useful skill, it is already part of ‘ascending a platform’ and therefore not needed to be tested separately. The item ‘avoids moving obstacles’ was suggested to be adapted into an item measuring the ability to perform a ‘sudden stop’ as this was seen to be more relevant for children.

### Conclusion phase 2: item selection of WMS for children

The WMS items which were deemed relevant by both the children or their parents and by the health care professionals were selected for further pilot testing in phase 3. The item ‘avoiding moving obstacles’ was adapted into ‘sudden stop’. Even though holding a wheelie was seen as not relevant by health care professionals, it was retained as a separate item as this WMS was regarded as highly relevant by children and their parents. This resulted in a 16 item WMS outcome measure, from here on called the UP-WMST.

### Results phase 3: Pilot testing of WMS items

One physiotherapist (30 years old, 4.5 years of experience) and one occupational therapist (28 years old, 5 years of experience) jointly administered the UP-WMST in eight children. All items were scored with an ability score (pass/fail) and a performance time score. The children’s age ranged from 5 to 11 years old, with five children diagnosed with Cerebral Palsy and three with other disabilities. The two health care professionals commented on the ease of administering the UP-WMST in an hour. For most items both health care professionals confirmed that the items had clear instructions and were easy to administer. However, the following items were less easy to administer. The dependability of weather conditions and the extra time burden of testing in- and outdoors made the outdoor items for rolling over soft surface ‘propel over grass’ and ‘propel over gravel’ too difficult to administer. The indoor item for rolling over soft surface ‘propel over a mat’ was retained. The material for the item ‘side slope’ was seen as too big and difficult to handle when setting up the test. Table 6 shows the remaining UP-WMST items after excluding the outdoor and side slope items. Therapists also suggested future changes in the scoring method of the items with a height difference. When a child passes the ability score, the quality of execution could be a more important indicator of the performance than the time it takes to complete the item.

**Table 6:** Selected items of the Utrecht Pediatric Wheelchair Mobility Skills Test after phase 3

Utrecht Pediatric Wheelchair Mobility Skills Test		Outcome parameter	
		Ability	Time
1. Propulsion forward^a, b, c, d^	10 m	Yes/No	Seconds
2. Propulsion backwards^a^	5 m	Yes/No	Seconds
3. Rolls on soft surface (mat)^a, c^	2 m	Yes/No	Seconds
4. Turns 90° while moving forward^a^	Left	Yes/No	Seconds
	Right	Yes/No	Seconds
5. Turns 90° while moving backward^a^	Left	Yes/No	Seconds
	Right	Yes/No	Seconds
6. Turns 180° in place^a^	Left	Yes/No	Seconds
	Right	Yes/No	Seconds
7. Sudden stop			Seconds
8. Opening/Closing a door^a, c^	Open toward	Yes/No	Seconds
	Open away	Yes/No	Seconds
9. Figure-of-8-shape^c^		Yes/No	Seconds
10. Holding a Wheelie^a, c^	30 s	Yes/No	Seconds
11. Slope ascent^a, c, d^	20%	Yes/No	Seconds
12. Slope descent^a, d^	20%	Yes/No	Seconds
13. Platform ascending^a, c, d^	5,10 cm	Yes/No	Seconds
14. Platform descending^a^	5,10 cm	Yes/No	Seconds
15. Doorstep^a, c^	2 cm	Yes/No	Seconds

^a^Wheelchair Skills Test, ^b^Wheelchair Propulsion Test, ^c^Wheelchair Circuit, ^d^5 Additional Mobility and Locomotor items

## Discussion

The objective of this article was to develop a WMS outcome measure for children. The results of the literature review in phase 1 are in accordance with previous systematic reviews [12, 14, 15] and show the wide range of available outcome measures used for assessing WMS. Only the TMT [43] was developed for children, but due to the small sample size (*n* = 11) this instrument was excluded for data synthesis and analysis. There are two WMS items in the TMT ‘propelling down the hall’ and ‘propelling up a ramp’. These two items are part of WMS outcome measures for adults and were therefore assessed on relevance in phase 2. No other WMS outcome measure has been developed or validated for children using a manual wheelchair. Furthermore none of the identified outcome measures showed good levels of evidence across all measurement properties. For example, most outcome measures showed a low level of evidence on content validity. Content validity is defined by COSMIN as ‘the degree to which the content of a measurement instrument is an adequate reflection of the construct to be measured’ [21]. Without good content validity, it is impossible to select the best outcome measure for a specific goal [51]. The construct the UP-WMST aims to assess skills of ‘wheelchair mobility’ as defined by the ICF d465 (http://apps.who.int/classifications/icfbrowser/). Phase 1 of this study shows that most existing WMS outcome measures do not assess ‘wheelchair mobility’ as defined by the ICF d465, but rather related concepts such as wheelchair user function or manual wheelchair use. While also important, these are different constructs and therefore only sections of these outcome measure were included that were relevant for assessing WMS.

In addition to assessing content validity and reliability, it is important to assess whether an outcome measure is responsive to detect change over time. The results of phase 1 showed limited levels of evidence on responsiveness available for only one outcome measure [31]. However, while there is no evidence regarding the responsiveness of the WST, the WST has been used in randomized controlled trials and seems responsive to measure change [6–9, 52]. Based on all the available psychometric data assessed, there was not a single WMS outcome measure suitable for validation in children. Therefore the second best option was to select outcome measures with some level of evidence on reliability and validity. The WST [20, 34], WPT [47], WC [30–33] and 5AML [42] already showed some level of evidence on an aspect of reliability and validity and both the WC and WST proved to be responsive to measure change in randomized controlled trials. The WMS items of these four outcome measures are the best available WMS items in current literature and were used or adapted for validation in children.

While there was little evidence available on the content validity of the identified WMS items, the results of phase 2 in this study show that most of the items on WMS were deemed as relevant by parents, children and health care professionals. These results can also be corroborated with a recent Delphi Survey [16], which reported on similar relevant items for a new WMS test for adults with an acute SCI. The only WMS items considered not relevant for children, were the more advanced WMS skills, such as descending or ascending stairs. Parents, children and health care professionals advised to include basic maneuvering tasks and include several height difference items for the outcome measure to be applicable for children. In contrast to adult manual wheelchair users, children are still developing as they grow older and different WMS will become more or less relevant. Furthermore, the size and weight of wheelchairs for young children are relatively large for their own size and strength, which makes it more difficult to execute WMS with height differences. When compared to WMS outcome measures for adults, it is important to include a higher proportion of more basic WMS in an outcome measure for children. The TMT [43] which was the only outcome measure developed for children contained two basic WMS items. These were ‘propelling down the hall’ and ‘propelling up a ramp’, and four other skills such as donning clothes or transfers. Similar adaptations were made in the WST 3.2 by Sawatsky et al. [10] for a pilot of WMS training in six children. They decreased the level of difficulty of the WST 3.2 by lowering the level change and incline for application in children. Even though outcome measures for wheelchair mobility had not been validated before in children, this study shows that most items related to basic wheelchair mobility with acceptable level of evidence in adults were considered relevant for assessment in children. Therefore the UP-WMST can be seen as an adapted version of adult outcome measures, specifically aimed at assessing basic wheelchair mobility, excluding more advanced items and items assessing different domains of the ICF (http://apps.who.int/classifications/icfbrowser/). However, as was mentioned by Sawatsky et al. [10] and confirmed by parents, children and health care professionals in this study, there could be a group of children with a high level of WMS and a basic WMS outcome measure might show a ceiling effect for these children. More advanced WMS are already assessed in WMS outcome measures for adults, but these outcome measures have not yet been validated for children. For example, TOWM and Wheelie test [38] consist of more advanced items with high platforms (20 cm) and several WMS skills in wheelie position. Future research should be aimed at developing and validating a similar advanced WMS outcome measure for children.

In addition to selecting relevant WMS items for children, it is also important to evaluate the applicability of the outcome measure in clinical practice. As suggested by Kirby [53] there are more assessment criteria which are useful to assess when selecting an outcome measure, such as time burden, availability of materials and ease of administering the test. Therefore, we examined the feasibility of administering the UP-WMST in phase 3 of this study. While the outdoor items were previously seen as relevant in phase 2, they were excluded from the UP-WMST after the results of phase 3 due to time burden of testing both in- and outdoors. When developing a more advanced test for wheelchair mobility in children, these outdoor items should be reconsidered for inclusion. Results of phase 3 also showed the need for an additional outcome parameter for the height difference items. All items are currently assessed on performance time and ability. The combination of these two outcome parameters seem to be in line with recent findings by Sawatzky et al. [54] that propulsion speed and ability are related. However, according to the results of phase 3, a more extensive scoring method should be included for the height difference items. Such a method could include a five point scoring method as used in the TOWM and Wheelie test [38], a performance score as used in the WC [31], or a safety score as described in the WST [35]. We are currently continuing with the validation of the UP-WMST and development of a qualitative scoring method which is able to distinguish between beginner or more advanced execution methods on an item.

This study was limited to WMS items with good measurement properties available in current literature. Surprisingly there was not one WMS outcome measure available with good levels of evidence across all measurement properties. The second best option was to select the best available WMS outcome measures for adults with some level of evidence across reliability and validity. The levels of evidence of these selected WMS items for responsiveness, minimal detectable change and minimal important change remain unknown. The feasibility of the UP-WMST was assessed by two health care professionals from the same rehabilitation center. It would be interesting to assess if the administration of the UP-WMST in a different setting or with different health care professionals would lead to the same results. Before the UP-WMST can be used in clinical practice, additional research towards responsiveness, interpretability, reliability and construct validity of the newly developed UP-WMST is warranted. Furthermore, the necessity of including basic WMS items could have been enhanced by the sampling of children and their parents used in this study for relevance checking. Children were recruited from a voluntary wheelchair mobility skills training program and interviewed a few weeks before the start of the program. Therefore, their level of wheelchair mobility could have been lower and this could have resulted in some bias towards more basic WMS. At the same time this is the group of children who attend a wheelchair skill training program and therefore the group of children the UP-WMST is developed for. Nevertheless, interviews only took place before the start of the wheelchair skill training program and children and parents might have underestimated the possible WMS a child is able to learn. Therefore, future research should evaluate possible ceiling effects of the UP-WMST.

## Conclusion

No single WMS outcome measure with good levels of evidence across all measurement properties was available for validation in children. However, four outcome measures did show levels of evidence on reliability and validity. The individual WMS items of these four outcome measure is the best knowledge available from literature and were used for relevance checking and validation in children. Parents, children using a manual wheelchair and health care professionals agreed on the necessity of including more basic WMS in an outcome measure for children compared to adults. The resulting 15 item UP-WMST outcome measure is easy to administer and demonstrates content validity for assessing WMS in children using a manual wheelchair. While this is the first step towards developing a WMS outcome measure for children, further assessment of reliability, construct validity and responsiveness is needed.

## Additional file

Additional file 1:
**Appendix 1.** Search filter for PubMed. Description of the search filter used in PubMed. (DOCX 11 kb)

## Abbreviations

5-AML: Five Additional Mobility and Locomotor Items
AM WC: Adapted Manual Wheelchair Circuit
COSMIN: COnsensus-based Standards for the selection of health Measurement INstruments
FIM: Functional Indepence Measure
HMAT: Harvey Mobility Assessment Tool
ICF: International Classification of Functioning, Disability and Health
JdG: Janke De Groot
LdG: Laura De Groot
MS: Marleen Sol
OCAWUP: Obstacle Course Assessment of Wheelchair User Performance
OV: Olaf Verschuren
SCI: Spinal cord injury
TM: Measurement of Time
TMT: Timed Motor Test for wheelchair users
TOMP: Tufts Assessment of Motor Performance
TOWM: Test of Wheeled Mobility
UP-WMST: Utrecht Pediatric Wheelchair Mobility Skills Test
VFM: Valutazione Funzionale Mielolesi
WAIMS: Wheelchair Assessment Instrument for people with Multiple Sclerosis
WC: Wheelchair Circuit
WC-PFP: Wheelchair physical functional performance test
WMS: Wheelchair mobility skills
WPT: Wheelchair Propulsion Test
WST: Wheelchair Skills Test
WUFA: Wheelchair Users Functional Assessment
