# Supplementary material for: Development of a wheelchair mobility skills test for children and adolescents: combining evidence with clinical expertise
Source: BMC Pediatr. 2017 Feb 13;17:51. doi: 10.1186/s12887-017-0809-9 (PMC5307781; doi:10.1186/s12887-017-0809-9)
Supplement: Additional file 1: — Appendix 1. Search filter for PubMed. Description of the search filter used in PubMed. (DOCX 11 kb) [file 12887_2017_809_MOESM1_ESM.docx]

**Appendix 1**

Search filter PUBMED

Search (((wheelchair AND rehabilitation AND mobility)) OR (wheelchair AND measurement AND assessment)) OR ((Wheelchair AND Mobility AND Skill) OR (Wheelchair AND Mobility AND Task) OR (Wheelchair AND Mobility AND Measurement) OR (Wheelchair AND Mobility AND Test) OR (Wheelchair AND Mobility AND ADL) OR (Wheelchair AND Mobility AND Functional) OR (Wheelchair AND Mobility AND Instrument) OR (Wheelchair AND Mobility AND Performance) OR (Wheelchair AND Mobility AND SCI) OR (Wheelchair AND Mobility AND Validity) OR (Wheelchair AND Mobility AND Reliability) OR (Wheelchair AND Mobility AND Pathology) OR (Wheelchair AND Mobility AND Behavior) OR (Wheelchair AND Mobility AND Activity) OR (Wheelchair AND Mobility AND Disability) OR (Wheelchair AND Mobility AND Assessment) OR (Wheelchair AND Mobility AND Quality of life)) Filters: Publication date from 2010/01/01
